# Supplementary material for: A Critical View of the Application of the APEX Software (Aqueous Photochemistry of Environmentally-Occurring Xenobiotics) to Predict Photoreaction Kinetics in Surface Freshwaters
Source: Molecules. 2019 Dec 18;25(1):9. doi: 10.3390/molecules25010009 (PMC7017383; doi:10.3390/molecules25010009)
Supplement: Supplementary file 1 [file molecules-25-00009-s001.zip › 3.2.4_gcc-4.4.0.zip!/3.2.4_gcc-4.4.0/3.2.4_gcc-4.4.0/doc/octave/html/interpreter/Manipulating-the-load-path.html]

Manipulating the load path - Untitled


Next: Subfunctions,
Up: Function Files


---

#### 11.7.1 Manipulating the load path

When a function is called, Octave searches a list of directories for
a file that contains the function declaration. This list of directories
is known as the load path. By default the load path contains
a list of directories distributed with Octave plus the current
working directory. To see your current load path call the `path`
function without any input or output arguments.

It is possible to add or remove directories to or from the load path
using `addpath` and `rmpath`. As an example, the following
code adds ~/Octave to the load path.

```
     addpath("~/Octave")
```

After this the directory ~/Octave will be searched for functions.

— Built-in Function: **addpath** (dir1, ...)  
— Built-in Function: **addpath** (dir1, ..., option)  
> Add dir1, ... to the current function search path. If
> option is "-begin" or 0 (the default), prepend the
> directory name to the current path. If option is "-end"
> or 1, append the directory name to the current path.
> Directories added to the path must exist.
>
>
>
> **See also:** path, rmpath, genpath, pathdef, savepath, pathsep.

— Built-in Function: **genpath** (dir)  
> Return a path constructed from dir and all its subdirectories.

— Built-in Function: **rmpath** (dir1, ...)  
> Remove dir1, ... from the current function search path.
>
>
>
> **See also:** path, addpath, genpath, pathdef, savepath, pathsep.

— Function File: **savepath** (file)  
> Save the portion of the current function search path, that is
> not set during Octave's initialization process, to file.
> If file is omitted, ~/.octaverc is used. If successful,
> `savepath` returns 0.
>
>
>
> **See also:** path, addpath, rmpath, genpath, pathdef, pathsep.

— Built-in Function: **path** (...)  
> Modify or display Octave's load path.
>
> If nargin and nargout are zero, display the elements of
> Octave's load path in an easy to read format.
>
> If nargin is zero and nargout is greater than zero, return the
> current load path.
>
> If nargin is greater than zero, concatenate the arguments,
> separating them with `pathsep()`. Set the internal search path
> to the result and return it.
>
> No checks are made for duplicate elements.
>
>
>
> **See also:** addpath, rmpath, genpath, pathdef, savepath, pathsep.

— Function File: val = **pathdef** ()  
> Return the default path for Octave.
> The path information is extracted from one of three sources.
> In order of preference, those are;
>
> 1. ~/.octaverc- <octave-home>/.../<version>/m/startup/octaverc- Octave's path prior to changes by any octaverc.
>
>
>
>
> **See also:** path, addpath, rmpath, genpath, savepath, pathsep.

— Built-in Function: val = **pathsep** ()  
— Built-in Function: old\_val = **pathsep** (new\_val)  
> Query or set the character used to separate directories in
> a path.
>
>
>
> **See also:** filesep, dir, ls.

— Built-in Function: **rehash** ()  
> Reinitialize Octave's load path directory cache.

— Built-in Function: **file\_in\_loadpath** (file)  
— Built-in Function: **file\_in\_loadpath** (file, "all")  
> Return the absolute name of file if it can be found in
> the list of directories specified by `path`.
> If no file is found, return an empty matrix.
>
> If the first argument is a cell array of strings, search each
> directory of the loadpath for element of the cell array and return
> the first that matches.
>
> If the second optional argument `"all"` is supplied, return
> a cell array containing the list of all files that have the same
> name in the path. If no files are found, return an empty cell array.
>
>
>
> **See also:** file\_in\_path, path.

— Built-in Function: **restoredefaultpath** (...)  
> Restore Octave's path to it's initial state at startup.
>
>
>
> **See also:** path, addpath, rmpath, genpath, pathdef, savepath, pathsep.

— Built-in Function: **command\_line\_path** (...)  
> Return the command line path variable.
>
>
>
> **See also:** path, addpath, rmpath, genpath, pathdef, savepath, pathsep.

— Built-in Function: **find\_dir\_in\_path** (dir)  
> Return the full name of the path element matching dir. The
> match is performed at the end of each path element. For example, if
> dir is `"foo/bar"`, it matches the path element
> `"/some/dir/foo/bar"`, but not `"/some/dir/foo/bar/baz"`
> or `"/some/dir/allfoo/bar"`.
